# Hydrogen Peroxide Flushes for Necrotizing Pancreatitis in a High‐Risk Surgical Patient: A Pathway for Durable Response Without Additional Endoscopic Intervention

**DOI:** 10.1155/crgm/5520452

**Published:** 2025-12-28

**Authors:** Smriti Kochhar, Ibrahim Yaghnam, Kofi Clarke

**Affiliations:** ^1^ Department of Medicine, Division of Internal Medicine, Penn State Milton S. Hershey Medical Center, Hershey, Pennsylvania, USA, pennstatehershey.org; ^2^ Department of Medicine, Division of Gastroenterology and Hepatology, Penn State Milton S. Hershey Medical Center, Hershey, Pennsylvania, USA, pennstatehershey.org; ^3^ Division of Gastroenterology, Banner Health, University of Arizona, Tucson, Arizona, USA, arizona.edu

**Keywords:** hydrogen peroxide, necrotizing pancreatitis, percutaneous drains, walled-off necrosis

## Abstract

Necrotizing pancreatitis (NP) is characterized by severe pancreatic inflammation with necrosis and a systemic inflammatory response. We describe the case of a 32‐year‐old female with NP successfully treated with a modified protocol using hydrogen peroxide (H_2_O_2_) flushes performed through percutaneous drains (PCDs). She was deemed high risk for endoscopic necrosectomy due to her significant cardiorespiratory comorbidities. As such H_2_O_2_‐assisted necrosectomy involving H_2_O_2_ instillation into the necrotic collections via the PCDs was performed. Sequential close clinical follow‐up to 1 year showed a durable response without adverse effects. To our knowledge, an extended, durable clinical response has not been previously reported. Further research is required to define clear guidelines on dosing, administration regimen, and follow‐up on PCD drainage.

## 1. Introduction

Severe acute pancreatitis (AP) is characterized by intense pancreatic inflammation and persistent organ failure. Up to 20% of patients with AP develop necrotizing pancreatitis (NP), historically associated with up to a 30% mortality rate [1]. NP often leads to prolonged ICU stays and may require invasive interventions for both local and systemic complications. More than two‐thirds of AP‐related deaths result from sepsis and organ failure [2]. Management of infected pancreatic necrosis with open surgical necrosectomy and antibiotics is associated with poorer outcomes [1]. Endoscopic necrosectomy is an effective approach with reduced mortality.

We present a case of NP with a complex clinical course, successfully managed using a modified drainage protocol involving hydrogen peroxide (H_2_O_2_) flushes through percutaneous drains (PCDs).

## 2. Case Report

**Figure 1:**
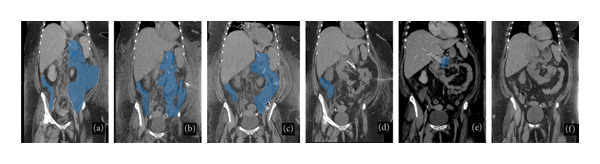
Progression of mixed density collections in the perigastric, left pericolic, and right pericolic region (shaded in blue) (a–d) throughout the treatment process. (a) Initial CT abdomen after 12‐Fr PCD. (b) 1 month after placement of 3 PCDs with significant ascites. (c, d) After H_2_O_2_ flush initiation with 1‐week and 1.5‐month follow‐up, respectively. (e) 3‐month follow‐up after treatment shows residual 3.7‐cm pancreatic cyst highlighted in blue. (f) S1‐year follow‐up with no residual disease.

A 32‐year‐old female patient with a past medical history including morbid obesity (BMI of 55) developed a bile leak following laparoscopic cholecystectomy and underwent endoscopic retrograde cholangiopancreatography (ERCP) with papillotomy and biliary stent placement. Her clinical course was complicated by post‐ERCP pancreatitis and acute respiratory distress syndrome requiring prolonged ventilator support and eventual tracheostomy placement. In addition, she developed bilateral pulmonary emboli requiring anticoagulation. Her course was further complicated by hemorrhagic pancreatitis, necessitating cessation of anticoagulation and placement of an inferior vena cava filter. Despite broad‐spectrum antibiotics and antifungals, she continued to be febrile for over 3 months. The clinical picture and imaging were consistent with extensive infected pancreatic necrosis and bilateral pericolic fluid collections. Due to her unstable condition, a 12‐French (Fr) PCD was placed into the 16.4 × 10.3 cm left perigastric collection (Figure 1(a)). She was transferred to our center for further management, including evaluation for possible endoscopic necrosectomy. The perigastric drain was upsized to 16 Fr because of ongoing fevers and concern for catheter occlusion, which resulted in transient unsustained improvement. A second 12‐Fr PCD was then placed into the enlarging left pericolic gutter collection, but this also provided minimal additional benefit. Since the necrosum continued to drain inadequately, both drains were progressively upsized with the intention of performing endoscopic necrosectomy once she stabilized. The left perigastric drain was increased from 16 Fr to 18 Fr and then to 28 Fr, and the left retroperitoneal drain was upsized from 12 Fr to 28 Fr. Additional percutaneous catheters were placed, including a 14 Fr drain positioned in the dependent left paracolic gutter adjacent to the 28‐Fr catheter (Figure 1(b)). She was deemed high risk for endoscopic necrosectomy due to her significant cardiorespiratory comorbidities.

Two management options for the 13.9 × 8.8 cm (right) and 18.8 × 11.8 cm (left) pericolic collections were discussed by a multidisciplinary team as follows:

1. Percutaneous direct endoscopic necrosectomy in the operating room, acknowledging that this would be a challenging task due to her body habitus and significant cardiorespiratory comorbidities.
2. H_2_O_2_ instillation into the necrotic collections via the PCDs for hydrogen peroxide–assisted necrosectomy.

The second approach was selected. A total of 200 mL of 1% diluted hydrogen peroxide (H_2_O_2_) was instilled and reaspirated through the PCDs twice daily for 6 weeks. One week after initiating H_2_O_2_ therapy, an additional 14‐Fr catheter was placed adjacent to the perigastric collection to access a loculated cavity directly. The aspirated fluid gradually transitioned from brown purulent material to tan, cloudy effluent as necrotic debris cleared. Both 14‐Fr multipurpose drains were connected to constant suction drainage via an accordion drain, while the two 28‐Fr Thal drains were maintained on gravity drainage.

All drains were confirmed to be in continuity via a small channel. The patient demonstrated gradual symptomatic improvement, with resolution of fever and leukocytosis. Follow‐up CT imaging showed progressive reduction in collection size at 1 week (Figure 1(c)) and further improvement at 1 month (Figure 1(d)). After 45 days of therapy, the drains were removed, and imaging confirmed near‐complete resolution of the necrosis. The patient was discharged to a rehabilitation facility without PCDs or tracheostomy.

**Figure 2:**
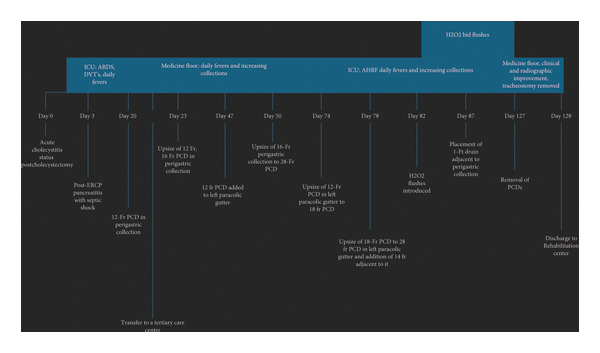
Timeline of the patient’s stay from initial PCD placement to removal with improvement in the clinical picture. ICU: intensive care unit; ARDS: acute respiratory distress syndrome; PCD: percutaneous drain; DVT: deep vein thrombosis; H_2_O_2_: hydrogen peroxide; AHRF: acute hypoxic respiratory failure.

At a 3‐month follow‐up, CT imaging confirmed persistent resolution of the walled‐off necrosis, and the patient remained clinically well (Figures 1(e) and 1(f)). Refer to Figure 2 for a timeline of drain placement and upsizing.

## 3. Discussion

Endoscopic necrosectomy is a widely accepted and preferred treatment modality for infected pancreatic necrosis. The “step‐up approach” beginning with PCD, has been shown to significantly reduce the morbidity and mortality associated with traditional open necrosectomy via laparotomy [3].

A major drawback of PCD as a definitive therapy has been the challenges associated with of solid/semisolid necrosum, which frequently leads to catheter blockage and limits efficacy. Alternative approaches using hydrogen peroxide–assisted or streptokinase necrosectomy have been described with positive results [1].

Previous studies have described chemical and mechanical necrosectomy in contrast to the approach used in our patient with hydrogen peroxide–assisted necrosectomy through PCD alone [4, 5]. There are currently no established protocols for H_2_O_2_ concentration, dosing, or volume during PCD. Prior reports describe various formulations, often including 3% H_2_O_2_ diluted with sterile saline at a 2:1 or 3:1 ratio [4]. We opted to use existing case reports to guide dosing and the instillation technique in our patient.

Messallam et al. reported a clinical success rate of 93.8% for endoscopic necrosectomy in conjunction with H_2_O_2_ compared to 78.9% in the standard group [6]. A separate systematic review and meta‐analysis of patients treated with necrosectomy and H_2_O_2_ lavage reported a clinical success rate of 91.6% and an adverse event rate of 19.3%. Reported complications included bleeding (7.9%), stent migration (11.3%), perforation (5.4%), infection (5.7%), and pulmonary adverse events (2.9%) [7].

However, in the context of percutaneous catheter drainage, Bhargava et al. reported significantly higher complication and mortality rates with H_2_O_2_ compared to streptokinase [2]. All patients in their study were surgical candidates at enrollment, with approximately 50% ultimately requiring surgery, and 50% died from complications. Table 1 summarizes studies that evaluate hydrogen peroxide–assisted necrosectomy techniques for the management of pancreatic necrosis [2, 6–9].

**Table 1:** Summary of studies evaluating hydrogen peroxide–assisted necrosectomy techniques for the management of pancreatic necrosis.

Study	Type of study	Population	Intervention	Comparator	Outcomes
Messallam et al. [6]	Retrospective cohort	204 Patients with walled‐off pancreatic necrosis	Direct endoscopic necrosectomy with hydrogen peroxide	Standard endoscopic necrosectomy	Higher clinical success (93.8% vs. 78.9%, *p* = 0.002), shorter time to resolution, similar complication rates
Bhargava et al. [2]	Randomized pilot study	30 Patients with infected pancreatic necrosis	Percutaneous catheter drainage with hydrogen peroxide	Streptokinase	Higher bleeding complications (20% vs. 6.6%), higher need for surgery (73% vs. 33.3%), higher mortality (60% vs. 33%)
Mohan et al. [7]	Systematic review and meta‐analysis	186 Patients with walled‐off pancreatic necrosis	Hydrogen peroxide–assisted endoscopic necrosectomy	None	High technical success (95.8%), high clinical success (91.6%), adverse events (19.3%)
Günay et al. [8]	Retrospective cohort study	24 Patients with walled‐off pancreatic necrosis	Hydrogen peroxide–assisted endoscopic necrosectomy	Standard endoscopic necrosectomy	Similar procedural success, fewer endoscopic interventions needed (4.2 vs. 6.1, *p* = 0.01)
Garg et al. [9]	Systematic review and meta‐analysis	454 Patients with walled‐off pancreatic necrosis	Hydrogen peroxide–assisted endoscopic necrosectomy	None	High technical success (97.3%), high clinical success (89.8%), adverse events (17.9%)

At 1 year posttreatment, no procedure‐related adverse events were reported in our patient. To our knowledge, the durability of response to H_2_O_2_ washout has not been described in previous reports.

Our case demonstrates that H_2_O_2_‐based chemical necrosectomy via PCD alone can be a viable alternative in selected high‐risk patients. However, further studies are needed to establish standardized protocols and assess long‐term outcomes for this approach.

## Consent

Informed patient consent was obtained for publication of the case details.

## Disclosure

All authors agree to be accountable for all aspects of the work in ensuring that questions related to the accuracy or integrity of any part of the work are appropriately investigated and resolved.

## Conflicts of Interest

The authors declare no conflicts of interest.

## Author Contributions

S.K. and I.Y.: conception and design of the work; acquisition, analysis, and interpretation of data for the work; and drafting of the manuscript. K.C.: analysis and interpretation of case and critical review for important intellectual content.

## Funding

This research received no specific grant from any funding agency in the public, commercial, or not‐for‐profit sectors.

## Data Availability

Data sharing is not applicable to this article as it is based on a single patient case report and does not involve a dataset.
